# Supplementary material for: Could hand-eye laterality profiles affect sport performance? A systematic review
Source: PeerJ. 2022 Nov 17;10:e14385. doi: 10.7717/peerj.14385 (PMC9676015; doi:10.7717/peerj.14385)
Supplement: Supplemental Information 1 [file peerj-10-14385-s001.docx]

**Appendix 1. Search strategy**

**Table A1.1. Search strategy for PsycINFO by EBSCOhost**

| Step | Search string |
| --- | --- |
| 1 | ("eye-hand dominance" OR "eye-hand laterality" OR "hand-eye dominance" OR "hand-eye laterality" OR "crossed laterality" OR "cross laterality" OR "contralateral dominance" OR "crosslateral dominance" OR "cross lateral dominance" OR "homogeneous laterality" OR "homogeneous dominance" OR "homogeneous preference" OR "ipsilateral preference" OR "ipsilateral dominance" OR "cross dominance" OR "crossed dominance" OR "hand-eye coordination" OR "eye-hand-coordination" OR "hand-eye preference" OR "eye-hand preference" OR "lateral preference" OR "lateral dominance" OR "cross-lateral dominance" OR "eyedness" OR "ocular dominance" OR "dominant eye" OR "ocular laterality" OR "handedness" OR "uncrossed dominance" OR "eye-hand coordination" OR "hand-eye preference" OR "eye-hand preference" OR "cross laterality" OR "uncrossed laterality" OR "left-handed" OR "left-handedness" OR "right-handed" OR "oculo-motor coordination") |
| 2 | "sport*" |
| 3 | #1 AND #2 |
| 4 | #3 AND LA (English OR Spanish OR French) |
| 5 | #4 AND PO (Human) |
| 6 | #5 AND PT (Journal) |

**Table A1.2. Search strategy for Medline by PUBMED**

| Step | Search string |
| --- | --- |
| 1 | ("eye-hand dominance" OR "eye-hand laterality" OR "hand-eye dominance" OR "hand-eye laterality" OR "crossed laterality" OR "cross laterality" OR "contralateral dominance" OR "crosslateral dominance" OR "cross lateral dominance" OR "homogeneous laterality" OR "homogeneous dominance" OR "homogeneous preference" OR "ipsilateral preference" OR "ipsilateral dominance" OR "cross dominance" OR "crossed dominance" OR "hand-eye coordination" OR "eye-hand-coordination" OR "hand-eye preference" OR "eye-hand preference" OR "lateral preference" OR "lateral dominance" OR "cross-lateral dominance" OR "eyedness" OR "ocular dominance" OR "dominant eye" OR "ocular laterality" OR "handedness" OR "uncrossed dominance" OR "eye-hand coordination" OR "hand-eye preference" OR "eye-hand preference" OR "cross laterality" OR "uncrossed laterality" OR "left-handed" OR "left-handedness" OR "right-handed" OR "oculo-motor coordination") |
| 2 | "sport*" |
| 3 | #1 AND #2 |
| 4 | #3 AND (english [Filter] OR spanish[Filter] OR french[Filter]) |
| 5 | #4 AND humans[Filter] |

**Table A1.3. Search strategy for Scopus by Elsevier**

| Step | Search string |
| --- | --- |
| 1 | ("eye-hand dominance" OR "eye-hand laterality" OR "hand-eye dominance" OR "hand-eye laterality" OR "crossed laterality" OR "cross laterality" OR "contralateral dominance" OR "crosslateral dominance" OR "cross lateral dominance" OR "homogeneous laterality" OR "homogeneous dominance" OR "homogeneous preference" OR "ipsilateral preference" OR "ipsilateral dominance" OR "cross dominance" OR "crossed dominance" OR "hand-eye coordination" OR "eye-hand-coordination" OR "hand-eye preference" OR "eye-hand preference" OR "lateral preference" OR "lateral dominance" OR "cross-lateral dominance" OR "eyedness" OR "ocular dominance" OR "dominant eye" OR "ocular laterality" OR "handedness" OR "uncrossed dominance" OR "eye-hand coordination" OR "hand-eye preference" OR "eye-hand preference" OR "cross laterality" OR "uncrossed laterality" OR "left-handed" OR "left-handedness" OR "right-handed" OR "oculo-motor coordination" ) |
| 2 | "sport*" |
| 3 | #1 AND #2 |
| 4 | #3 AND ( LIMIT-TO ( LANGUAGE,"English" ) OR LIMIT-TO ( LANGUAGE,"French" ) OR LIMIT-TO ( LANGUAGE,"Spanish" ) ) |
| 6 | #4 AND ( LIMIT-TO ( SRCTYPE , "j" ) ) |

**Table A1.4. Search strategy for Dissertations & Theses Global by ProQuest**

| Step | Search string |
| --- | --- |
| 1 | "eye-hand dominance" OR "eye-hand laterality" OR "hand-eye dominance" OR "hand-eye laterality" OR "crossed laterality" OR "cross laterality" OR "contralateral dominance" OR "crosslateral dominance" OR "cross lateral dominance" OR "homogeneous laterality" OR "homogeneous dominance" OR "homogeneous preference" OR "ipsilateral preference" OR "ipsilateral dominance" OR "cross dominance" OR "crossed dominance" OR "hand-eye coordination" OR "eye-hand-coordination" OR "hand-eye preference" OR "eye-hand preference" OR "lateral preference" OR "lateral dominance" OR "cross-lateral dominance" OR "eyedness" OR "ocular dominance" OR "dominant eye" OR "ocular laterality" OR "handedness" OR "uncrossed dominance" OR "eye-hand coordination" OR "hand-eye preference" OR "eye-hand preference" OR "cross laterality" OR "uncrossed laterality" OR "left-handed" OR "left-handedness" OR "right-handed" OR "oculo-motor coordination" |
| 2 | "sport*" |
| 3 | #1 AND #2 |
| 4 | #3 AND limits applied (English OR Spanish OR French) AND (Doctoral dissertations) AND (Full text) |
| 5 | #4 AND limits applied (publication date from 2010) |

**Table A1.5. Search strategy for GOOGLE (grey literature)**

| Search string |
| --- |
| eye-hand laterality OR hand-eye laterality OR oculomotor laterality AND sport filetype:pdf |
