# Supplementary material for: Could hand-eye laterality profiles affect sport performance? A systematic review
Source: PeerJ. 2022 Nov 17;10:e14385. doi: 10.7717/peerj.14385 (PMC9676015; doi:10.7717/peerj.14385)
Supplement: Supplemental Information 2 [file peerj-10-14385-s002.docx]

Appendix 2. Risk of bias of reviewed studies.

Table A2.1. JBI critical appraisal tool for analytical Cross Sectional Studies.

| Authors | Q1 | Q2 | Q3 | Q4 | Q5 | Q6 | Q7 | Q8 | %YES |
| --- | --- | --- | --- | --- | --- | --- | --- | --- | --- |
| Dalton, Guillon & Naroo., (2015) | – | + | + | + | – | – | + | + | 62.5% |
| Mann, Runswick & Allen (2016) | – | + | + | + | – | – | + | + | 62.5% |
| Laborde et al. (2009). | – | – | + | + | – | – | + | + | 50% |
| Nosek, Hurdálková, & Cihlář. (2018). | – | – | NA | + | – | – | + | + | 37.5% |
| Portal & Romano (1998). | – | + | – | – | – | – | NA | + | 25% |
| Pointer (2008). | – | – | – | – | – | – | + | + | 25% |
| Quevedo et al. (2014). | – | + | – | – | – | – | + | – | 25% |
| Shick, J (1977). | – | + | – | – | – | – | + | + | 37.5% |
| Shick, J (1971). | – | + | – | – | – | – | + | + | 37.5% |
| Sugiyama & Lee. (2005) | – | + | + | + | + | – | + | + | 75% |
| Zouhal H et al. (2018) | + | + | + | + | + | – | + | + | 87.5% |

Q1. Were the criteria for inclusion in the sample clearly defined? Q2. Were the study subjects and the settings described in detail? Q3. Was the exposure measured in a valid and reliable way? Q4. Were objective, standard criteria used for measurement of the condition? Q5. Were confounding factors identified? Q6. Were strategies to deal with confounding factors stated? Q7. Were the outcomes measured in a valid and reliable way? Q8. Was appropriate statistical analysis used?; +: Yes; –: No; ?: Unclear; NA: not applicable

Table A2.2. JBI critical appraisal tool for prevalence studies.

| Authors | Q1 | Q2 | Q3 | Q4 | Q5 | Q6 | Q7 | Q8 | Q9 | %YES |
| --- | --- | --- | --- | --- | --- | --- | --- | --- | --- | --- |
| ﻿Ziagkas et al. (2018) | + | + | + | – | – | – | + | – | + | 55,5% |

Q1. Was the sample frame appropriate to address the target population? Q2. Were study participants sampled in an appropriate way? Q3. Was the sample size adequate? Q4. Were the study subjects and the setting described in detail? Q5. Was the data analysis conducted with sufficient coverage of the identified sample? Q6. Were valid methods used for the identification of the condition? Q7. Was the condition measured in a standard, reliable way for all participants? Q8. Was appropriate statistical analysis used? Q9. Was the response rate adequate, and if not, was the low response rate managed appropriately? + - Yes; – - No; U-Unclear: Not/Applicable

Table A3.3. JBI critical appraisal tool for quasi experimental studies.

| Authors | Q1 | Q2 | Q3 | Q4 | Q5 | Q6 | Q7 | Q8 | Q9 | %YES |
| --- | --- | --- | --- | --- | --- | --- | --- | --- | --- | --- |
| Lopez-Diaz et al. (2015) | + | – | + | – | + | – | + | + | + | 66,67% |
| Razeghi et al. (2012) | + | – | + | – | + | – | + | + | + | 66,67% |

Q1. Is it clear in the study what is the ‘cause’ and what is the ‘effect’ (i.e. there is no confusion about which variable comes first)? Q2. Were the participants included in any comparisons similar? Q3. Were the participants included in any comparisons receiving similar treatment/care, other than the exposure or intervention of interest? Q4. Was there a control group? Q5. Were there multiple measurements of the outcome both pre and post the intervention/exposure? Q6. Was follow up complete and if not, were differences between groups in terms of their follow up adequately described and analyzed? Q7. Were outcomes measured in a reliable way? Q8. Was appropriate statistical analysis used? Q9. Was appropriate statistical analysis used?+ - Yes; – - No; U-Unclear: Not/Applicable
