# Supplementary material for: Could hand-eye laterality profiles affect sport performance? A systematic review
Source: PeerJ. 2022 Nov 17;10:e14385. doi: 10.7717/peerj.14385 (PMC9676015; doi:10.7717/peerj.14385)
Supplement: Supplemental Information 3 [file peerj-10-14385-s003.docx]

1.    The rationale for conducting the systematic review / meta-analysis.

There are no published systematic reviews that bring together the different percentages and performance effects analyzed in different sports to cover the objectives of our study: a) estimate the prevalence of hand-eye laterality profiles, b) examine the relationship between hand-eye laterality profiles, psychological factors and sports performance, and c) propose a methodological and terminological consensus. The few literature reviews in the field are very old or only partially cover our objectives.

2.    The contribution that it makes to knowledge in light of previously published related reports, including other meta-analyses and systematic reviews.

Previous systematic reviews have attempted to determine the percentage of C-HELP and UC-HELP in the normal population (Bourassa, Bryden & MacManus, 1996; MacManus et al., 1999). But these are revisions from more than twenty years ago. An updated systematic review is needed to complete the studies that have linked some performance indicators to the hand-eye laterality profile with specific sports such as tennis (Ziagkas, Mavvidis & Georgios, 2018), golf (Dalton, Guillon & Naroo, 2015; Sugiyama & Lee, 2005), baseball (Laby et al., 1998; Classe et al., 1996; Portal & Romano, 1998), cricket (Thomas, Harden & Rogers, 2005) or basketball (Shick, 1971, 1977; Lopez-Diaz et al., 2015).

Thus, our review is necessary for providing information that could help shape future research in this area. For example, at the applied level, our review will reveal that certain sports have a different prevalence of hand-eye laterality profiles than the normal population and that these specific profiles may indicate some benefits for players in some sports. We also propose a standardized terminology and protocol for hand-eye laterality assessment in sports, and we intend to clarify the prevalence of hand-eye laterality profiles in the normal population.

**References**

Classe, J. G., Daum, K., Semes, L., Wisniewski, J., Rutstein, R., Alexander, L., … Bartolucci, A. (1996). Association between eye and hand dominance and hitting, fielding and pitching skill  among players of the Southern Baseball League. *Journal of the American Optometric Association*, *67*(2), 81–86.

Dalton, K., Guillon, M., & Naroo, S. A. (2015). Ocular dominance and handedness in golf putting. *Optometry and Vision Science*, *92*(10), 968–975. DOI: <https://doi.org/10.1097/OPX.0000000000000690>

Sugiyama, Y., & Lee, M.S. (2005). Relation of Eye Dominance With Performance and Subjective Ratings in Golf Putting. *Perceptual and Motor Skills*, *100*(3), 761–766. DOI: <https://doi.org/10.2466/PMS.100.3.761-766>

Shick, J. (1971). Relationship between depth perception and hand-eye dominance and free-throw shooting in college women. *Perceptual and Motor Skills*, *33*(2), 539–542. DOI: <https://doi.org/10.2466/pms.1971.33.2.539>

Shick, J. (1977). Relationship between hand eye dominance and lateral errors in basketball free throw shooting. *Perceptual and Motor Skills*, *44*(2), 549–550. DOI: <https://doi.org/10.2466/pms.1977.44.2.549>

Thomas, N. G., Harden, L. M., & Rogers, G. G. (2005). Visual evoked potentials, reaction times and eye dominance in cricketers. *Journal of Sports Medicine and Physical Fitness*, *45*(3), 428–433. DOI: <https://doi.org/10.1016/j.soilbio.2015.08.008>

Laby, D. M., Kirschen, D. G., Rosenbaum, A. L., & Mellman, M. F. (1998). The effect of ocular dominance on the performance of professional baseball players. *Ophthalmology*, *105*(5), 864–866. DOI: <https://doi.org/10.1016/S0161-6420(98)95027-8>

Lopez-Diaz, C. J., Niño García, N., Sillero Quintana, M., & Lorenzo Calvo, A. (2015). ¿Puede el principio de lateralidades múltiples mejorar el porcentaje de acierto en el tiro a canasta? [May the multiple dominances principle improve the percentage average in the basketball shot?]. *Cuadernos de Psicología Del Deporte*, *15*(3), 211–218. DOI: <https://doi.org/10.4321/S1578-84232015000300024>

Portal, J. M., & Romano, P. E. (1988). Patterns of Eye–Hand Dominance in Baseball Players. *New England Journal of Medicine*, *319*(10), 655–656. DOI: <https://doi.org/10.1056/NEJM198809083191018>

Ziagkas, E., Mavvidis, A., & Georgios, D. (2017). Investigating the role of ipsilateral and contralateral eye-hand dominance in tennis serve accuracy of amateur tennis players. *Journal of Physical Education and Sport*, *17*(2), 867–870. DOI: https://doi.org/10.7752/jpes.2017.02132
